# Pan‐cancer analysis of TIM‐3 transcriptomic expression reveals high levels in pancreatic cancer and interpatient heterogeneity

**DOI:** 10.1002/cam4.6844

**Published:** 2023-12-22

**Authors:** Jungah Lim, Razelle Kurzrock, Daisuke Nishizaki, Hirotaka Miyashita, Jacob J. Adashek, Suzanna Lee, Sarabjot Pabla, Mary Nesline, Jeffrey M. Conroy, Paul DePietro, Scott M. Lippman, Shumei Kato

**Affiliations:** ^1^ National Medical Center Seoul South Korea; ^2^ MCW Cancer Center Milwaukee Wisconsin USA; ^3^ WIN Consortium Paris France; ^4^ University of Nebraska Omaha Nebraska USA; ^5^ Center for Personalized Cancer Therapy and Division of Hematology and Oncology, Department of Medicine, UC San Diego Moores Cancer Center La Jolla California USA; ^6^ Dartmouth Cancer Center, Hematology and Medical Oncology Lebanon New Hampshire USA; ^7^ Department of Oncology, The Sidney Kimmel Comprehensive Cancer Center The Johns Hopkins Hospital Baltimore Maryland USA; ^8^ OmniSeq Inc. Buffalo New York USA

## Abstract

**Background:**

T‐cell immunoglobulin and mucin domain‐containing protein 3 (TIM‐3), an immune checkpoint receptor, dampens immune function. TIM‐3 antagonists have entered the clinic.

**Methods:**

We analyzed TIM‐3 transcriptomic expression in 514 diverse cancers. Transcript abundance was normalized to internal housekeeping genes and ranked (0–100 percentile) to a reference population (735 tumors; 35 histologies [high≥75 percentile rank]). Ninety tumors (17.5%) demonstrated high TIM‐3 expression.

**Results:**

TIM‐3 expression varied between and within tumor types. However, high TIM‐3 expression was more common in pancreatic cancer (20/55 tumors, 36.4%; odds ratio, 95% confidence interval (pancreatic vs. other tumors) = 3.176 (1.733–5.818; *p* < 0.001, multivariate]). High TIM‐3 also significantly and independently correlated with high PD‐L1 (*p* = 0.014) and high CTLA‐4 (*p* < 0.001) transcriptomic expression (multivariate).

**Conclusions:**

These observations indicate that TIM‐3 RNA expression is heterogeneous, but more common in pancreatic cancer and in tumors exploiting PD‐L1 and CTLA‐4 checkpoints. Clinical trials with patient selection for matched immune‐targeted combinations may be warranted.

## 1 INTRODUCTION

**FIGURE 1:**
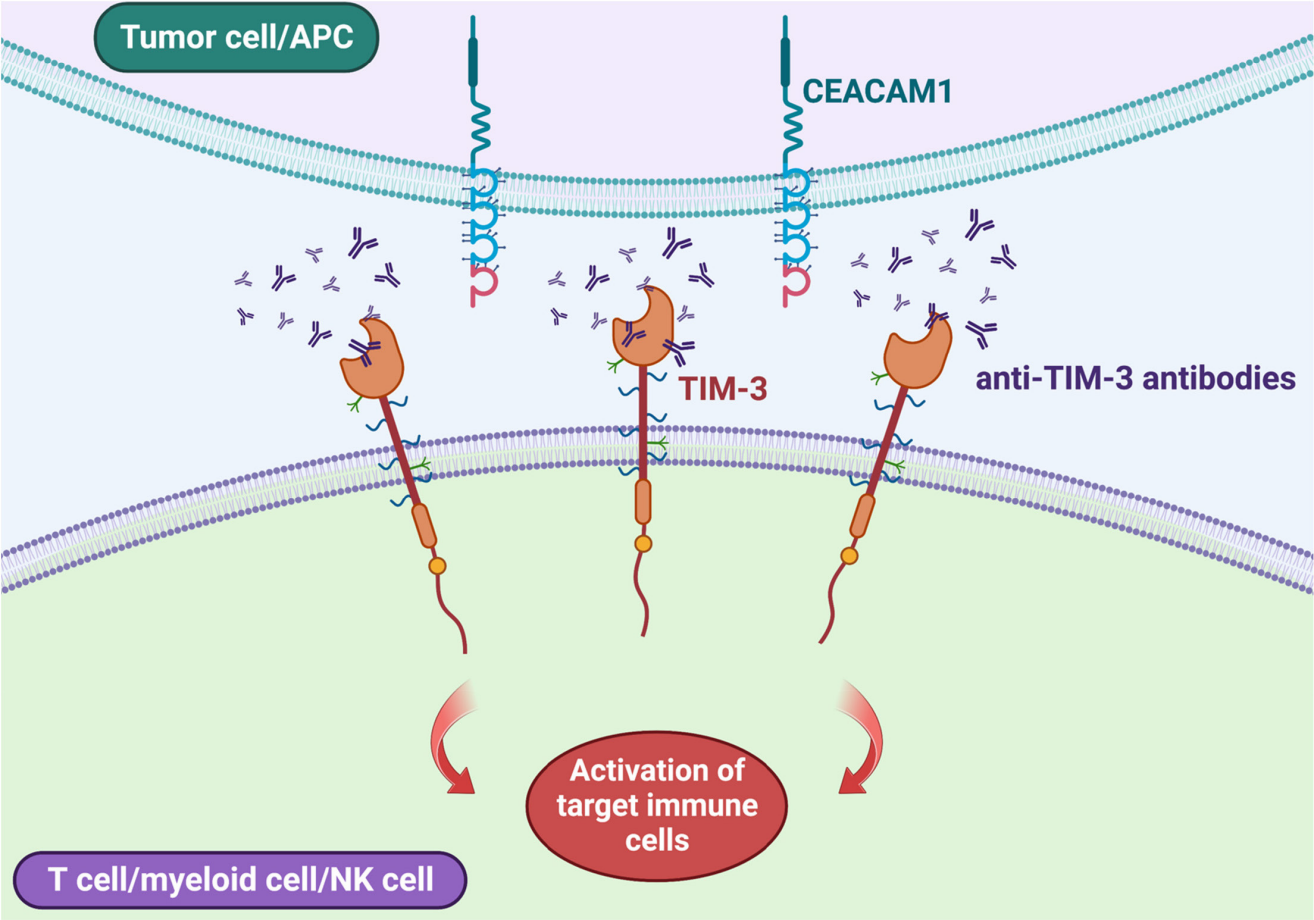
Interaction of TIM‐3, anti‐TIM‐3 antibodies, and tumor cells or antigen presenting cells (APCs). Schematic of a tumor cell or APC and an immune cell (T cell, myeloid cell, or NK cell) and the effect of anti‐TIM‐3 antibodies allowing for maturation and activation of the target immune cell. TIM‐3 can be found on T cells, myeloid cells, and NK cells and functions as an inhibitory receptor to suppress activity of these target immune cells. CEACAM1 acts on APCs and some tumor cells to endow the inhibitory function of TIM‐3 on various immune cells.

T‐cell immunoglobulin and mucin domain‐containing protein 3 (TIM‐3), an immune checkpoint receptor, is present in various immune cells and is known to suppress immune function.^1^, ^2^, ^3^, ^4^, ^5^ TIM‐3 is expressed on several immune cells such as cytotoxic T cells, regulatory T cells, natural killer (NK) cells, and some antigen‐presenting cells including dendritic cells (DCs). Under regular circumstances, TIM‐3 inhibits both innate and adaptive immune cells. TIM‐3 can inhibit effector T cells by activating suppressor cells such as Treg cells and DCs. TIM‐3 also plays a role in T‐cell and NK‐cell exhaustion (Figure 1). Moreover, TIM‐3 is often co‐expressed and exerts its inhibitory function with other immune checkpoint receptors (e.g., PD‐1, LAG‐3, and TIGIT) as a modulator on CD4^+^ and CD8^+^ T cells.^1^, ^2^, ^3^, ^4^, ^5^

Since TIM‐3 functions as an immune suppressor, therapeutically, inhibition of TIM‐3 would modulate antitumor immune responses by activating multiple immune cells.^1^ Indeed, TIM‐3 can inhibit antitumor immunity by mediating T‐cell exhaustion, and blockade of TIM‐3 pathway, which can lead to enhanced antitumor immunity by an increase in the production of interferon‐gamma (IFN‐γ) in T cells.^6^ As TIM‐3 is capable of suppressing various innate immune cells, it is not surprising that cancer hijacks this system to evade immune surveillance. Upregulation of TIM‐3 has been reported in solid tumors including non‐small cell lung cancer (NSCLC), hepatocellular carcinoma, colorectal cancer, cervical cancer, ovarian cancer, head and neck cancer, and in hematologic tumors such as acute myelogenous leukemia, breast cancer, and non‐Hodgkin lymphoma.^7^, ^8^, ^9^, ^10^, ^11^, ^12^, ^13^, ^14^, ^15^, ^16^ Moreover, high TIM‐3 was identified as a potential predictor of poor outcome after anti‐PD‐1/PD‐L1 in some of those cancers, perhaps because the tumors take advantage of the TIM‐3 checkpoint to inactivate the immune system.^17^ One study found that, in patients with pancreatic cancer, there was a lack of correlation between T‐cell TIM3 immunohistochemistry (IHC) expression and patient prognosis; however, patients in this study did not receive anti‐PD‐1/PD‐L1, anti‐CTLA‐4, nor anti‐TIM‐3 treatments.^18^ Overall, TIM‐3 is being recognized as a promising new therapeutic target in cancer immunotherapy.

Checkpoint blockade, especially with PD‐1/PD‐L1 inhibitors, has changed the clinical course of diverse cancer types; however, overall response rates are about 20% across cancers.^19^ Even so, with appropriate markers such as high microsatellite instability, high tumor mutational burden (TMB), mismatch repair gene deficiency, and high PD‐L1, the response rate can be 50%–60%.^19^, ^20^, ^21^, ^22^ Therefore, the investigation of markers to predict the response is important.

Several studies suggest that a combination approach, including chemotherapy, radiation, targeted therapies, and other immune modulators, may optimize immunotherapy effectiveness.^23^, ^24^ Since TIM‐3 is an important checkpoint and also known to be a potential resistant marker for PD‐1/PD‐L1 inhibitors,^17^ there are now many clinical trials evaluating the efficacy of TIM‐3 inhibitors (Table 1). However, most studies do not require a biomarker (such as TIM‐3 expression status) for patient selection.^20^

**TABLE 1:** Examples of clinical trials evaluating TIM‐3 inhibitors.

Drug	Combination	Phase	TIM‐3 expression	Cancer type	Results	NCT number
Sym023	Single agent	I	Not required for enrollment	Solid cancers and lymphoma	*N* = 24, clinical outcomes NA	NCT03489343
Sym023	Plus anti‐PD‐1, anti‐LAG‐3, Irinotecan	Ib	Not required for enrollment	Biliary tract cancer	*N* = 100, clinical outcomes NA	NCT04641871
MBG453	Plus anti‐PD‐1	I‐Ib/II	Not required for enrollment	Advanced solid cancers	*N* = 252, Clinical outcomes NA	NCT02608268
MBG453	Single agent	II	Not required for enrollment	Lower risk MDS	*N* = 20, clinical outcomes NA	NCT04823624
MBG453	Plus NIS793^a^, Canakinumab^b^	Ib	Not required for enrollment	Lower risk MDS	*N* = 90, clinical outcomes NA	NCT04810611
MBG453	Plus HDM201^c^, Venetoclax^d^	Ib	Not required for enrollment	AML, MDS	*N* = 80, Clinical outcomes NA	NCT03940352
MBG453	Plus Azacytidine, placebo	III	Not required for enrollment	MDS, CMML‐2	*N* = 50, clinical outcomes NA	NCT04266301
MBG453	Plus placebo, hypomethylating agent	II	Not required for enrollment	MDS	*N* = 127, clinical outcomes NA	NCT03946670
TSR‐022	Plus Nivolumab^e^ and chemotherapy	I	Not required for enrollment	Advanced solid cancer	*N* = 369, clinical outcomes not available	NCT02817633
TSR‐022	Plus Niraparib, Bevacizumab, chemotherapy, TSR042 (anti‐PD‐1)	Ib	Not required for enrollment	NSCLC	*N* = 58, clinical outcomes NA	NCT03307785
TSR‐022	Plus dostarlimab^f^ (TSR‐042)	II	Not required for enrollment	Liver cancer	*N* = 42, clinical outcomes NA	NCT03680508
TSR‐022	Plus dostarlimab^f^ (TSR‐042)	II	Not required for enrollment	Melanoma (Stage III–IV)	*N* = 56, clinical outcomes NA	NCT04139902
MBG453	Plus anti‐PD‐1, chemotherapy	Ib	Not required for enrollment	AML or high‐risk MDS	*N* = 243, clinical outcomes NA	NCT03066648
MBG453	Plus anti‐PD‐1 and stereotactic radiosurgery	I	Not required for enrollment	Glioblastoma multiforme	*N* = 15, clinical outcomes NA	NCT03961971
INCAGN02390	Plus anti‐PD‐1 and anti‐LAG‐3	I–II	Not required for enrollment	Advanced malignancies	*N* = 52, clinical outcomes NA	NCT04370704
INCAGN02390	Single agent	I	Not required for enrollment	Advanced cancer	*N* = 40, completed, but clinical outcomes NA	NCT03652077
LY3321367	Plus anti‐PD‐L1 antibody	I	Not required for enrollment	Advanced relapsed solid cancer	*N* = 275, ORR = 4%	NCT03099109^25^
LY3415244	PD‐L1/TIM‐3 bispecific antibody	I	Not required for enrollment	Advanced solid tumors	*N* = 12, terminated early due to immunogenicity (anaphylactic infusion reactions and treatment‐emergent antidrug antibodies	NCT03752177^26^
RO7121661	Plus anti‐PD‐1	I	Not required for enrollment	Advanced solid cancer	*N* = 134, Clinical outcomes NA	NCT03708328
AZD7789	Plus anti‐PD‐1	I–IIa	Not required for enrollment	NSCLC	*N* = 81, Clinical outcomes NA	NCT04931654
BGB‐A425	Plus Tislelizumab^g^	I–II	Not required for enrollment	HNSCC, NSCLC (I) RCC (II)	*N* = 162, Clinical outcomes NA	NCT03744468
R07121661	Plus anti‐PD‐1, anti‐LAG‐3, Nivolumab^e^	II	Not required for enrollment	Advanced esophageal cancer	*N* = 255, Clinical outcomes NA	NCT04785820
TQB2618	Single agent	I	Not required for enrollment	Advanced solid cancer	*N* = 50, Clinical outcomes NA	NCT04623892

Abbreviations: AML, acute myeloid leukemia; CMML, chronic myelomonocytic leukemia; HNSCC, head and neck squamous cell cancer; LAG, lymphocyte activation gene; MDS, myelodysplastic syndrome; ORR, objective response rate; NA, not available; NSCLC, non‐small cell lung cancer; RCC, renal cell cancer.

^a^ NIS793: Anti‐TGF‐β monoclonal antibody.

^b^ Kanakinumab: Anti‐IL‐1β monoclonal antibody.

^c^ HDM201: p53‐Mdm2 interaction inhibitor.

^d^ Venetoclax: Bcl‐2 inhibitor.

^e^ Nivolumab: anti PD‐1 antibody.

^f^ Dostarlimab: anti PD‐1 antibody (FDA approved for the treatment of uterine cancer).

^g^ Tislelizumab: anti PD‐1 antibody.

Herein, we examine the transcriptomic expression level of TIM‐3 in 514 patients with diverse cancers and demonstrate that TIM‐3 expression levels are variable across tumors, but are elevated more frequently in pancreatic adenocarcinomas as compared to other cancers, suggesting that TIM‐3 therapeutic targeting may be warranted in pancreatic malignancies.

## 2 METHODS

### 2.1 Patients

The RNA expression level of TIM‐3 along with PD‐L1, PD‐1, and CTLA‐4 in various types of advanced solid tumors from 514 patients seen at the University of California San Diego (UCSD) Moores Cancer Center for Personalized Therapy was analyzed at a Clinical Laboratory Improvement Amendments (CLIA)‐licensed and College of American Pathologist (CAP)‐accredited clinical laboratory, OmniSeq (https://www.omniseq.com/; Table S1). In addition to the expression data, the information on the patients' age, sex, histological types of primary cancer, and TMB were collected. If a patient had two or more different samples analyzed in different days, the one from earlier timepoint was used for the analysis. All studies were conducted following the guidelines of the UCSD Institutional Review Board for data collection (Study of Personalized Cancer Therapy to Determine Response and Toxicity, UCSD_PREDICT, NCT02478931) and any investigational interventions for which patients consented.

### 2.2 Sampling of tissue and analysis of cancer immunity markers

Following tissue collection, tumors were provided as formalin‐fixed, paraffin‐embedded (FFPE) samples, and evaluated by RNA sequence at OmniSeq laboratory. All RNA was extracted from FFPE using truXTRAC FFPE extraction kit (Covaris, Inc., Woburn, MA), with some modification to the manufacturer's instructions. After purification, RNA was dissolved in 50 μL water and the yield was measured through Quant‐iT RNA HS assay (Thermo Fisher Scientific, Waltham, MA). For appropriate library preparation, the predefined titer of 10 ng RNA was referred to as acceptance criteria. Torrent Suite's plugin immuneResponseRNA (v5.2.0.0) 34 was used for the absolute reading of the RNA sequence. The RNA expression of 397 different genes was measured.

Transcript abundance was normalized to internal housekeeping gene profiles and ranked (0–100 percentile) in a standardized manner to a reference population of 735 tumors including 35 histologies. The RNA expression profiles were stratified by rank values into “High” (75–100 percentile), “Intermediate” (25–74 percentile), and “Low” (0–24 percentile).

### 2.3 Definition of variables

For TMB, genomic DNA was extracted from qualified FFPE tumors (>30% neoplastic nuclei) by means of the truXTRAC FFPE extraction kit (Covaris) with 10 ng DNA input for library preparation. DNA libraries were prepared with Ion AmpliSeq targeted sequencing chemistry using the Comprehensive Cancer Panel, followed by enrichment and template preparation using the Ion Chef system, and sequencing on the Ion S5XL 540 chip (Thermo Fisher Scientific). Following the removal of germline variants, synonymous variants, indels, and single nucleotide variants (SNVs) with <5% variant allele fraction (VAF), TMB is reported as eligible mutations per qualified panel size (Mutations/Megabase).

### 2.4 Data analysis

We used logistic regression to perform univariate and multivariate analyses for high TIM‐3 expression. Variables that were significant in univariate analysis (*p* ≤ 0.05) were included in multivariate analysis. All the analyses were performed with the use of IBM SPSS Statistics version 28.

## 3 RESULTS

**FIGURE 2:**
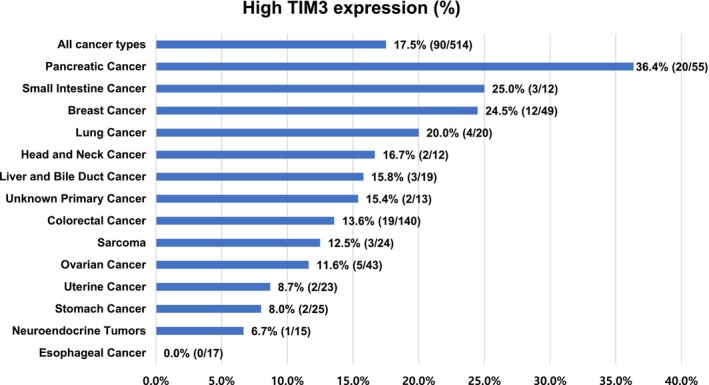
TIM‐3 transcriptomic expression according to cancer type. High expression was defined as ≥75 percentile transcriptomic rank as compared in standardized manner to a reference population of 735 tumors including 35 histologies (see also Section 2). Only cancers with at least 10 samples were included in this figure.

**FIGURE 3:**
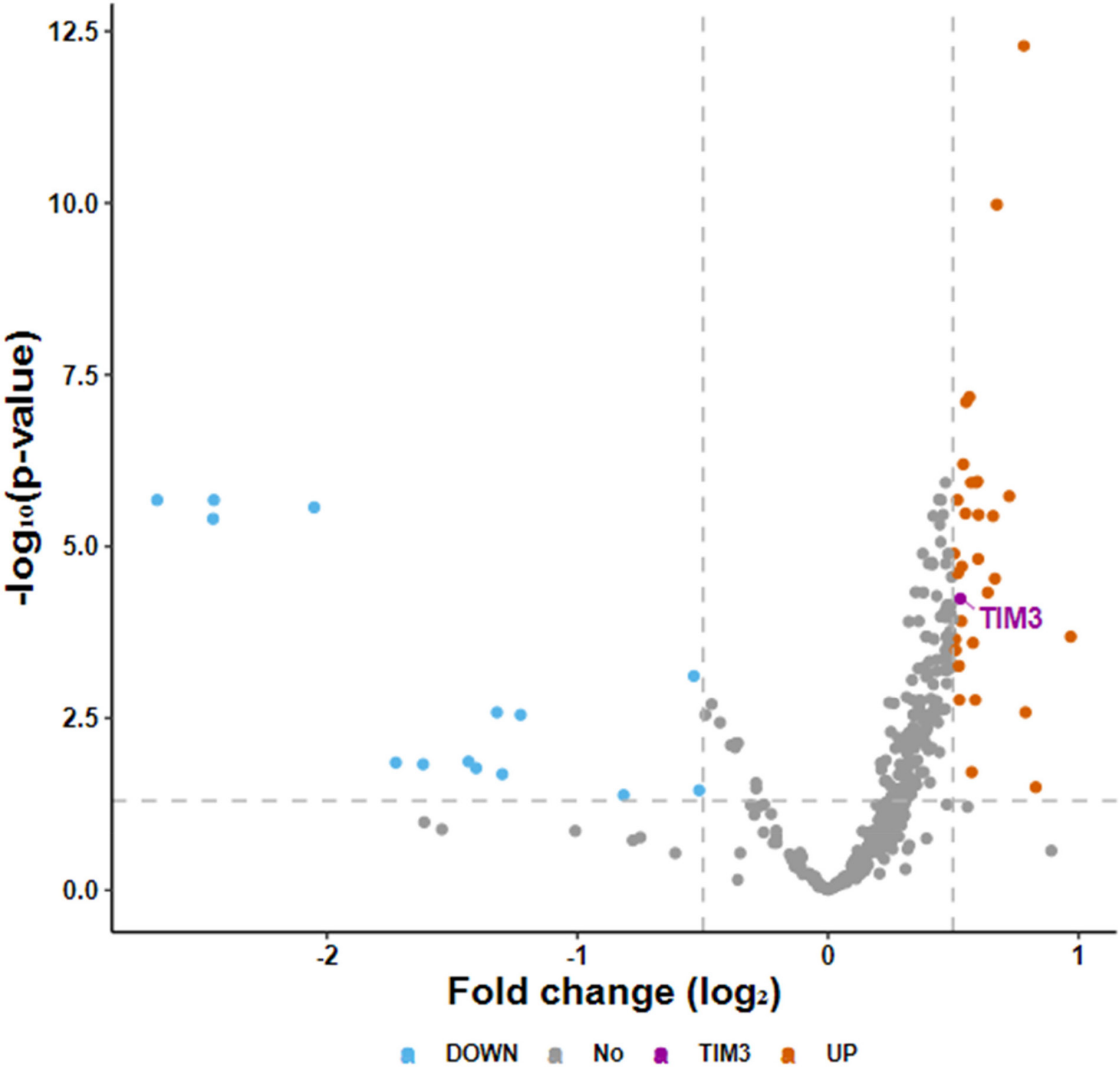
Volcano plot summarizing the expression rate. Among 397 different genes, TIM‐3 exhibited a 1.44‐fold change (0.53 when transformed by log2) with an adjusted p‐value of 0.0000573 (−4.24 when transformed by log10) in pancreatic cancer patients versus other tumors. Orange dots are high in pancreatic cancer and the purple dot represents TIM‐3, which is high. Therefore, TIM‐3 expression is high in pancreatic cancer compared to other cancers.

We investigated 514 patients with diverse cancers; 489 had confirmed metastatic or locally advanced disease. There were *N* = 204 (39.7%) men and *N* = 310 (60.3%) women. Median age was 61 years old (*N* = 256 were 61 years old or older and *N* = 258 were younger than 61 years old). The most common cancer types tested for TIM‐3 expression were colorectal cancer (*N* = 140) followed by pancreatic cancer (*N* = 55), breast cancer (*N* = 49), and ovarian cancer (*N* = 43). Among 514 patients, 90 patients (90/514, 17.5%) had high TIM‐3 expression (≥75 percentile RNA rank; Figure 2). We find that TIM‐3 RNA expression is more common in pancreatic cancer and tumors exploiting PD‐L1 and CTLA‐4 checkpoints. To check if TIM‐3 RNA expression is significantly higher in patients with pancreatic cancer, we depicted a volcano plot among 397 different genes. TIM‐3 exhibited a 1.44‐fold change (0.53 when transformed by log2) with an adjusted p‐value of 0.0000573 (−4.24 when transformed by log10; Figure 3).

### 3.1 High TIM‐3 RNA expression was more common in pancreatic cancer than in other tumor types

Among diverse cancer types, TIM‐3 overexpression was most commonly seen in pancreatic cancer (20/55, 36.4%) followed by small intestine cancer (3/12, 25%), breast cancer (12/49, 24.5%), and lung cancer (4/20, 20%; Figure 2). The percentage of patients with high TIM‐3 expression was significantly higher in patients with pancreatic cancer than in those with non‐pancreatic cancer (20/55, 36.4% vs. 70/459, 15.3%, *p* < 0.001 [multivariate]; Table 2). The percentage of patients with high TIM‐3 expression in colorectal, breast, and ovarian cancers was not significantly different from those in non‐colorectal, non‐breast, and non‐ovarian cancers, respectively (Table 2, Figure 2). Overall, RNA expression level of TIM‐3 was individually variable both across and within malignancies.

**TABLE 2:** Factors associated with high TIM‐3 expression.

	High TIM‐3^a^ (*N* = 90)	Odds ratio (95% CI)	Univariate, *p* value	Multivariate, *p* value^b^
Men (*N* = 204)	Men (*N* = 33/204, 16.2%)	0.857 (0.535–1.371)	0.519	
Women (*N* = 310)	Women (*N* = 57/310, 18.4%)			
Age ≥median (61 years) (*N* = 256) and <median (*N* = 258)	Age ≥median (*N* = 42/256, 16.4%) Age <median (*N* = 48/258, 18.6%)	0.859 (0.544–1.354)	0.512	
*Tumor types*				
Colorectal adenocarcinoma (*N* = 140)	Colorectal (*N* = 19/140,13.6%)	0.670 (0.387–1.160)	0.151	
Non‐colorectal (*N* = 374)	Non colorectal (*N* = 71/374, 19.0%)			
Pancreatic adenocarcinoma (*N* = 55)	Pancreatic (*N* = 20/55, 36.4%)	3.176 (1.733–5.818)	<0.001	<0.001
Non‐pancreatic (*N* = 459)	Non pancreatic (*N* = 70/459, 15.3%)			
Breast adenocarcinoma (*N* = 49)	Breast (*N* = 12/49, 24.5%)	1.609 (0.803–3.224)	0.176	
Non‐breast (*n* = 465)	Non breast (*N* = 78/465, 16.8%)			
Ovarian (*N* = 43)	Ovarian (*N* = 5/43, 11.6%)	0.598 (0.228–1.563)	0.289	
Non‐ovarian (*N* = 471)	Non ovarian (*N* = 85/471, 18.0%)			
TMB ≥10 mutations/mb (*N* = 33)	High TMB (*N* = 6/33, 18.2%)	1.272 (0.505–3.208)	0.609	
Not high TMB (<10 mutations/mb) (*N* = 417)	Not high TMB (*N* = 62/417, 14.9%)			
High PD‐1 (*N* = 93)^a^	High PD‐1 (*N* = 34/93, 36.6%)	3.756 (2.262–6.237)	<0.001	0.255
Not high PD‐1 (*N* = 421)	Not high PD‐1 (*N* = 56/421, 13.3%)			
High PD‐L1 (*N* = 67)^a^	High PD‐L1 (*N* = 26/67, 38.8%)	3.795 (2.172–6.631)	<0.001	0.014
Not high PD‐L1 (*N* = 447)	Not high PD‐L1 (*N* = 64/447, 14.3%)			
High CTLA‐4 (*N* = 87)^a^	High CTLA‐4 (N = 39/87, 44.8%)	5.990 (3.583–10.014)	<0.001	<0.001
Not High CTLA‐4 (*N* = 427)	Not high CTLA‐4 (N = 51/427, 11.9%)			

^a^ High CTLA‐4, High PD‐1, High PD‐L1, High TIM‐3 ≥75 percentile RNA rank.

^b^ *p* values that were significant in univariate analysis (*p* ≤ 0.05) were included in multivariate analysis.

### 3.2 Overexpression of TIM‐3 transcripts was significantly associated with PD‐L1 and CTLA‐4 transcript overexpression

**FIGURE 4:**
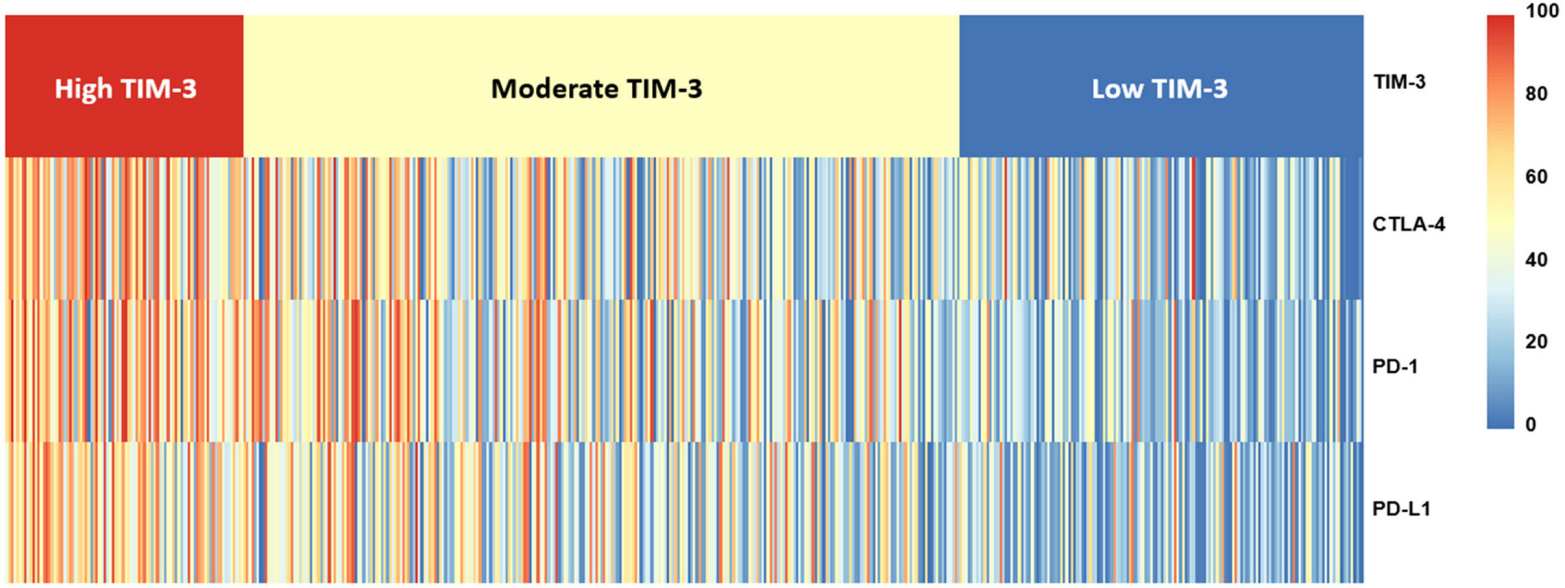
Heatmap depicting expression level of immune markers. This heat map depicts the expression levels of each immune marker (CTLA‐4, PD‐1, and PD‐L1) according to the TIM‐3 expression levels (low, moderate, and high; *N* = 514). In patients with high levels of TIM‐3, the expressions of CTLA‐4, PD‐1, and PD‐L1 were observed to be higher compared to patients with moderate or low expression levels of TIM‐3.

We then evaluated the TIM‐3 co‐expression pattern with other clinically important checkpoints (PD‐1, PD‐L1, and CTLA‐4). The percentage of patients with high TIM‐3 expression was significantly higher in high PD‐1 (odds ratio [OR]: 3.756, *p* < 0.001), high PD‐L1 (OR: 3.795, *p* < 0.001), and high CTLA‐4 (OR: 5.990, *p* < 0.001) groups than in non‐high PD‐1, non‐high PD‐L1, and non‐high CTLA‐4 groups, respectively (univariate analysis). Statistical significance for the association with high TIM‐3 remained in high PD‐L1 and high CTLA‐4 groups after multivariate analysis (*p* = 0.014 and *p* < 0.001, respectively; Table 2), further characterized by a heatmap (Figure 4). High TIM‐3 expression was not associated with high TMB (Table 2).

### 3.3 Validated in cBioportal public database: overexpression of TIM‐3 transcripts was significantly associated with PD‐L1 and CTLA‐4 transcript overexpression

We analyzed another pan‐cancer cohort (*N* = 570), which we obtained from cBioportal, to validate our finding that high TIM‐3 is associated with high CTLA‐4 and PD‐1. High CTLA‐4 (odds ratio 3.31; 95% CI, 1.83–5.94; *p* < 0.001) and high PD‐L1 (odd ratio 4.71; 95% CI, 2.79–7.95; *p* < 0.001) were also associated with high TIM‐3 in this cohort.

## 4 DISCUSSION

Most clinical trials conducted with TIM‐3 inhibitors in various types of cancers are still ongoing (Table 1). In one phase I study (NCT03099109), the TIM‐3 inhibitor (LY3321367) with or without PD‐L1 antibody was evaluated in patients with advanced solid tumors and the objective response rate was only 4% in the combination therapy cohort.^25^ NCT03752177 (LY3415244, a bispecific antibody against TIM‐3 and PD‐L1) terminated early (Table 1) during the dose escalation phase (*N* = 12 patients with advanced solid tumors) because of clinically significant anaphylactic infusion‐related reactions and treatment‐emergent antidrug antibodies.^26^ To our knowledge, there was no study that required a biomarker, such as expression of TIM‐3, as an inclusion criteria (Table 1).

In our current study, across cancer types, high TIM‐3 transcriptomic expression was found in only 17.5% of tumor samples. In esophageal cancer (0%), no TIM‐3 high expressors were identified. The next lowest expressing TIM3 cancers were neuroendocrine tumors (6.7%), stomach cancer (8%), and uterine cancer (8.7%). Pancreatic cancer, however, demonstrated high TIM‐3 RNA expression in 36.4% of cancers and this correlation was significant and independent in multivariate analysis. These data are consistent with prior studies showing high expression of TIM‐3 in pancreatic cancer.^27^ TIM‐3 IHC did not correlate with pancreatic cancer prognosis in prior studies.^18^ The next most common high TIM‐3 expressing histologies were small intestine cancer (25%), breast cancer (24.5%), and lung cancer (20%). It is, therefore, plausible that selection of patients by tumor TIM‐3 level may be important for response, and that pancreatic cancers should be a focus for clinical trials of TIM‐3 antagonists.^17^, ^20^

High TIM‐3 also correlated significantly and independently with high PD‐L1 and high CTLA‐4 expression, though not with high TMB (Table 2). Therefore, it may be that, in tumors that co‐express high TIM‐3 and high PD‐L1 or CTLA‐4, TIM‐3 inhibitors should be administered together with anti‐PD‐1/PD‐L1 and/or anti‐CTLA‐4 agents. It has been previously reported that a customized, matched gene‐targeted therapy approach (including with the use of transcriptomic biomarkers) can improve clinical outcomes, and it is plausible that a similar patient selection based on immune marker expression may be important for targeted immunotherapeutics as well.^28^, ^29^, ^30^

Our study has several limitations. First, the sample size is relatively small. Second, our study included diverse cancers, though the latter may point to the generalizability of the observations. Third, molecular/immune analysis was ordered by the treating physicians, thereby perhaps imposing a selection bias. Additional studies with larger sample sizes are needed to validate our findings. Another limitation is the lack of TIM‐3 protein expression, for example, flow cytometry data or IHC staining from slices of the paraffin‐embedded tumor samples. An additional limitation is that the data were obtained from whole tumor samples since TIM3 may be differentially expressed in different cell types.

In conclusion, our data suggest that TIM‐3 RNA expression is variable across and within malignancies and, therefore, individualized assessment of TIM‐3 transcriptomic level may be important. Even so, certain tumors have a significant and independent propensity to express high TIM‐3 transcript levels, including pancreatic cancer and tumors with high PD‐L1 and/or high CTLA‐4 transcript levels. Our observations require validation in additional cohorts. Moreover, future studies should examine correlation between TIM3 levels and outcome. Selection of patients by TIM‐3 levels, as well as levels of other checkpoints for TIM‐3 combination studies, merits investigation in clinical trials of TIM‐3 inhibitors.

## AUTHOR CONTRIBUTIONS

**Jungah Lim:** Formal analysis (equal); methodology (equal); writing – original draft (equal); writing – review and editing (equal). **Razelle Kurzrock:** Conceptualization (equal); visualization (equal); writing – original draft (equal); writing – review and editing (equal). **Daisuke Nishizaki:** Data curation (equal); formal analysis (equal); writing – review and editing (equal). **Hirotaka Miyashita:** Data curation (equal); writing – review and editing (equal). **Jacob J. Adashek:** Writing – review and editing (equal). **Suzanna Lee:** Data curation (equal); resources (equal); writing – review and editing (equal). **Sarabjot Pabla:** Methodology (equal); resources (equal); writing – review and editing (equal). **Mary Nesline:** Writing – review and editing (equal). **Jeffrey M. Conroy:** Writing – review and editing (equal). **Paul DePietro:** Writing – review and editing (equal). **Scott M. Lippman:** Supervision (equal); validation (equal); writing – review and editing (equal). **Shumei Kato:** Supervision (equal); validation (equal); visualization (equal); writing – original draft (equal); writing – review and editing (equal).

## CONFLICT OF INTEREST STATEMENT

Razelle Kurzrock has received research funding from Biological Dynamics, Boehringer Ingelheim, Debiopharm, Foundation Medicine, Genentech, Grifols, Guardant, Incyte, Konica Minolta, Medimmune, Merck Serono, Omniseq, Pfizer, Sequenom, Takeda, and TopAlliance; as well as consultant and/or speaker fees and/or advisory board for Actuate Therapeutics, AstraZeneca, Bicara Therapeutics, Biological Dynamics, Caris, Daiichi Sankyo, Inc., EISAI, EOM Pharmaceuticals, Iylon, Merck, NeoGenomics, Neomed, Pfizer, Prosperdtx, Roche, TD2/Volastra, Turning Point Therapeutics, X‐Biotech; has an equity interest in CureMatch Inc., CureMetrix, and IDbyDNA; serves on the Board of CureMatch and CureMetrix, and is a co‐founder of CureMatch. Jungah Lim, Hirotaka Miyashita, and Daisuke Nishizaki have no conflicts of interest. Jacob J. Adashek serves on the advisory board of CureMatch Inc. Suzanna Lee has no conflicts of interest. Sarabjot Pabla, Mary Nesline, Jeffrey M. Conroy, Paul DePietro are employees of Omniseq. Scott M. Lippman is the co‐founder of io9 and is on Biological Dynamics, Inc. Scientific Advisory Board. Shumei Kato serves as a consultant for Foundation Medicine. He receives speaker's fee from Roche and advisory board for Pfizer. He has research funding from ACT Genomics, Sysmex, Konica Minolta, and OmniSeq.

## Supporting information


Table S1.

## Data Availability

The datasets used and/or analyzed during the current study are available from the corresponding authors on reasonable request.
