# Supplementary material for: Pan‐cancer analysis of TIM‐3 transcriptomic expression reveals high levels in pancreatic cancer and interpatient heterogeneity
Source: Cancer Med. 2023 Dec 22;13(1):e6844. doi: 10.1002/cam4.6844 (PMC10807558; doi:10.1002/cam4.6844)
Supplement: Supplementary file 1 — Table S1. [file CAM4-13-e6844-s001.zip › legends.docx]

" Table S1 Caption: This table represents all of the patient included in the study: cancer diagnosis, age, sex, TIM-3 level, tumor mutation burden, PD-1 level, PD-L1 level, CTLA-4 level. "
