# Supplementary material for: Pan‐cancer analysis of TIM‐3 transcriptomic expression reveals high levels in pancreatic cancer and interpatient heterogeneity
Source: Cancer Med. 2023 Dec 22;13(1):e6844. doi: 10.1002/cam4.6844 (PMC10807558; doi:10.1002/cam4.6844)
Supplement: Supplementary file 1 — Table S1. [file CAM4-13-e6844-s001.zip › Supplemental Table 1 Transcriptome data March 25 2023.docx]

| **Cancer Diagnosis** | **Patient Age (years)** | **Patient Sex** | **TIM3*** | **TMB (mutations/megabase) Result** | **PD-1*** | **PD-L1*** | **CTLA-4*** |
| --- | --- | --- | --- | --- | --- | --- | --- |
| Ovarian Cancer | 69.59 | Female | 74 |  | 80 | 68 | 86 |
| Breast Cancer | 67.49 | Female | 49 | 5.2 | 33 | 94 | 69 |
| Sarcoma | 48.92 | Female | 17 | 3.5 | 8 | 0 | 8 |
| Thyroid Cancer | 61.46 | Male | 51 | 1.7 | 75 | 89 | 62 |
| Colorectal Cancer | 52.26 | Male | 42 | 5.2 | 26 | 48 | 63 |
| Melanoma | 33.32 | Male | 71 | 13 | 78 | 41 | 74 |
| Sarcoma | 31.03 | Female | 81 | 0.8 | 47 | 30 | 36 |
| Sarcoma | 56.62 | Female | 12 | 3.4 | 85 | 27 | 82 |
| Head and Neck Cancer | 58.67 | Female | 65 | 1.7 | 88 | 66 | 75 |
| Colorectal Cancer | 44.79 | Female | 96 | 2.6 | 46 | 48 | 49 |
| Sarcoma | 40.69 | Female | 11 | 3.5 | 9 | 14 | 5 |
| Gallbladder and Extrahepatic Bile Duct Cancers | 60.15 | Male | 53 | 5.2 | 92 | 58 | 76 |
| Ovarian Cancer | 63.2 | Female | 65 | 3.5 | 58 | 33 | 51 |
| Sarcoma | 43.12 | Male | 9 | 1.7 | 51 | 4 | 1 |
| Colorectal Cancer | 64.19 | Male | 61 | 4.3 | 60 | 65 | 81 |
| Adrenal Gland Cancer | 69.94 | Female | 26 | 2.6 | 5 | 2 | 19 |
| Prostate Cancer | 60.13 | Male | 55 | 0 | 20 | 41 | 26 |
| Pancreatic Cancer | 54.07 | Male | 9 | 1.7 | 3 | 3 | 0 |
| Pancreatic Cancer | 87.44 | Male | 74 |  | 33 | 81 | 23 |
| Colorectal Cancer | 64.95 | Female | 26 | 4.5 | 40 | 21 | 68 |
| Lung Cancer | 74.46 | Female | 72 | 4.3 | 86 | 92 | 89 |
| Breast Cancer | 39.15 | Female | 52 | 2.6 | 53 | 67 | 48 |
| Small Intestine Cancer | 74.91 | Male | 91 | 6.9 | 73 | 76 | 85 |
| Colorectal Cancer | 36.79 | Male | 95 |  | 54 | 60 | 80 |
| Colorectal Cancer | 72.02 | Female | 21 | 4.4 | 53 | 65 | 55 |
| Pancreatic Cancer | 49.89 | Male | 67 | 0 | 57 | 15 | 20 |
| Lung Cancer | 65.51 | Female | 44 | 1.7 | 0 | 4 | 2 |
| Stomach Cancer | 52.16 | Male | 37 | 4.3 | 90 | 57 | 79 |
| Colorectal Cancer | 35.26 | Female | 68 | 4.4 | 51 | 7 | 30 |
| Liver and Bile Duct Cancer | 59.75 | Female | 74 | 4.4 | 56 | 46 | 42 |
| Liver and Bile Duct Cancer | 61.62 | Female | 86 |  | 7 | 46 | 33 |
| Colorectal Cancer | 41.22 | Female | 97 |  | 72 | 65 | 70 |
| Colorectal Cancer | 36.68 | Male | 24 |  | 38 | 68 | 53 |
| Liver and Bile Duct Cancer | 58.03 | Male | 18 | 7.9 | 37 | 9 | 1 |
| Breast Cancer | 62.64 | Female | 56 | 1.7 | 32 | 16 | 4 |
| Colorectal Cancer | 60.68 | Female | 98 |  | 48 | 43 | 58 |
| Sarcoma | 73.87 | Female | 49 | 3.5 | 86 | 6 | 54 |
| Breast Cancer | 39.75 | Female | 80 |  | 59 | 53 | 55 |
| Head and Neck Cancer | 84.08 | Male | 6 | 7.9 | 14 | 12 | 18 |
| Ovarian Cancer | 72.28 | Female | 38 | 3.4 | 67 | 41 | 79 |
| Colorectal Cancer | 71.01 | Male | 38 | 7.9 | 17 | 40 | 14 |
| Pancreatic Cancer | 74.44 | Male | 81 | 4.4 | 58 | 57 | 46 |
| Stomach Cancer | 74.62 | Female | 50 |  | 92 | 90 | 86 |
| Breast Cancer | 69.15 | Female | 68 | 7 | 32 | 51 | 19 |
| Pancreatic Cancer | 61.01 | Female | 76 |  | 79 | 42 | 54 |
| Esophageal Cancer | 54.59 | Male | 7 | 3.5 | 12 | 29 | 16 |
| Esophageal Cancer | 63.85 | Male | 13 |  | 40 | 74 | 57 |
| Female Genital Cancer | 64.6 | Female | 84 | 10.5 | 99 | 54 | 97 |
| Breast Cancer | 56.55 | Female | 60 | 16.7 | 71 | 9 | 37 |
| Liver and Bile Duct Cancer | 80.19 | Female | 17 |  | 9 | 23 | 16 |
| Pancreatic Cancer | 74.22 | Female | 1 | 2.6 | 3 | 20 | 23 |
| Pancreatic Cancer | 86.74 | Female | 92 | 6.9 | 46 | 71 | 44 |
| Colorectal Cancer | 54.9 | Male | 11 |  | 42 | 34 | 36 |
| Lung Cancer | 40.29 | Male | 91 | 1.7 | 18 | 39 | 76 |
| Basal Cell Carcinoma of the Skin | 59.9 | Male | 26 |  | 37 | 52 | 68 |
| Small Intestine Cancer | 81.1 | Male | 43 | 10.5 | 0 | 37 | 0 |
| Head and Neck Cancer | 40.51 | Female | 92 | 5.2 | 41 | 55 | 21 |
| Colorectal Cancer | 40.46 | Male | 0 | 12.6 | 0 | 1 | 0 |
| Stomach Cancer | 45.69 | Female | 81 | 1.7 | 35 | 13 | 27 |
| Esophageal Cancer | 67 | Male | 68 | 6.2 | 63 | 59 | 47 |
| Colorectal Cancer | 26.15 | Male | 15 | 4.3 | 20 | 80 | 31 |
| Pancreatic Cancer | 76.37 | Female | 92 | 0 | 61 | 68 | 79 |
| Ovarian Cancer | 64.09 | Female | 45 | 5.3 | 15 | 46 | 34 |
| Liver and Bile Duct Cancer | 66.92 | Female | 60 | 6.9 | 15 | 22 | 15 |
| Retroperitoneum and Peritoneum Cancer | 73.92 | Female | 89 |  | 95 | 78 | 99 |
| Liver and Bile Duct Cancer | 59.76 | Female | 71 | 2.6 | 56 | 37 | 44 |
| Breast Cancer | 40.3 | Female | 48 | 1.7 | 24 | 17 | 37 |
| Colorectal Cancer | 79.61 | Female | 5 | 12.2 | 23 | 14 | 44 |
| Gallbladder and Extrahepatic Bile Duct Cancers | 62.22 | Female | 83 |  | 82 | 56 | 91 |
| Head and Neck Cancer | 57.33 | Female | 10 | 2.6 | 3 | 9 | 7 |
| Colorectal Cancer | 53.84 | Female | 62 |  | 12 | 16 | 49 |
| Adrenal Gland Cancer | 37.07 | Female | 36 | 0 | 2 | 2 | 12 |
| Thymic Cancer | 27.48 | Male | 11 | 1.7 | 92 | 88 | 0 |
| Unknown Primary Cancer | 67.87 | Male | 32 | 3.4 | 0 | 53 | 1 |
| Ovarian Cancer | 52.33 | Female | 45 | 2.7 | 26 | 33 | 38 |
| Ovarian Cancer | 70.35 | Female | 12 | 0.8 | 15 | 37 | 24 |
| Breast Cancer | 63.93 | Female | 57 | 2.6 | 52 | 67 | 49 |
| Colorectal Cancer | 32.01 | Male | 3 | 5.3 | 20 | 48 | 33 |
| Stomach Cancer | 26.14 | Female | 64 | 2.6 | 24 | 75 | 54 |
| Ovarian Cancer | 80.96 | Female | 71 | 9.6 | 61 | 64 | 66 |
| Cervical Cancer | 50.45 | Female | 74 |  | 94 | 87 | 98 |
| Lung Cancer | 76.77 | Male | 95 | 1.7 | 91 | 88 | 90 |
| Colorectal Cancer | 65.12 | Male | 39 | 3.5 | 37 | 79 | 74 |
| Colorectal Cancer | 68.15 | Male | 87 | 11.5 | 73 | 74 | 68 |
| Colorectal Cancer | 69.12 | Male | 72 | 8.8 | 83 | 54 | 92 |
| Stomach Cancer | 66.97 | Male | 2 | 8.9 | 0 | 7 | 0 |
| Bladder Cancer | 74.18 | Male | 18 | 12.1 | 0 | 0 | 0 |
| Neuroendocrine Tumors | 37.37 | Female | 29 | 1.7 | 78 | 47 | 25 |
| Lung Cancer | 65.1 | Female | 0 | 6.2 | 0 | 15 | 5 |
| Colorectal Cancer | 49.49 | Female | 32 | 6.1 | 47 | 32 | 54 |
| Lung Cancer | 32.92 | Male | 31 | 5.2 | 20 | 35 | 5 |
| Pancreatic Cancer | 64.31 | Female | 55 | 5 | 36 | 55 | 41 |
| Cervical Cancer | 30.51 | Female | 32 | 7.9 | 59 | 88 | 56 |
| Neuroendocrine Tumors | 39.92 | Male | 2 | 5.2 | 6 | 21 | 39 |
| Sarcoma | 72.23 | Female | 34 | 1.7 | 32 | 5 | 12 |
| Ovarian Cancer | 68.91 | Female | 24 | 0.8 | 26 | 42 | 47 |
| Ovarian Cancer | 64.87 | Female | 16 | 2.6 | 51 | 66 | 43 |
| Colorectal Cancer | 56.84 | Female | 57 | 6.1 | 39 | 37 | 38 |
| Ovarian Cancer | 74.27 | Female | 4 | 6.1 | 19 | 31 | 9 |
| Bladder Cancer | 54.04 | Male | 69 | 4.3 | 47 | 57 | 70 |
| Small Intestine Cancer | 50.07 | Female | 49 |  | 38 | 21 | 44 |
| Colorectal Cancer | 54.43 | Female | 76 | 2.6 | 46 | 31 | 55 |
| Colorectal Cancer | 56.09 | Male | 55 | 8.7 | 44 | 39 | 76 |
| Head and Neck Cancer | 46.54 | Male | 76 | 6.1 | 42 | 32 | 18 |
| Uterine Cancer | 70.8 | Female | 64 | 24.3 | 85 | 53 | 88 |
| Sarcoma | 54.07 | Female | 59 | 1.7 | 47 | 3 | 0 |
| Stomach Cancer | 82.76 | Male | 42 | 6 | 47 | 29 | 32 |
| Colorectal Cancer | 64.07 | Female | 51 | 18.3 | 74 | 87 | 74 |
| Colorectal Cancer | 50.08 | Female | 7 | 7.8 | 0 | 7 | 6 |
| Uterine Cancer | 68.52 | Female | 9 | 4.3 | 53 | 31 | 49 |
| Colorectal Cancer | 67.7 | Male | 35 | 1.7 | 39 | 28 | 48 |
| Colorectal Cancer | 61.36 | Female | 46 | 3.4 | 10 | 33 | 13 |
| Eye and Adnexa Cancers | 52.95 | Male | 39 | 0 | 8 | 11 | 13 |
| Ovarian Cancer | 39.61 | Female | 21 | 3.5 | 82 | 8 | 13 |
| Uterine Cancer | 57.73 | Female | 6 | 3.4 | 3 | 26 | 11 |
| Sarcoma | 68.22 | Male | 4 | 2.6 | 19 | 6 | 50 |
| Esophageal Cancer | 69.56 | Female | 12 | 7 | 24 | 47 | 50 |
| Lung Cancer | 67.42 | Female | 30 | 12.2 | 73 | 11 | 82 |
| Ovarian Cancer | 72.85 | Female | 36 | 1.7 | 39 | 50 | 47 |
| Pancreatic Cancer | 76.92 | Male | 33 |  | 14 | 14 | 13 |
| Pancreatic Cancer | 57.24 | Male | 77 | 5.2 | 35 | 85 | 42 |
| Uterine Cancer | 79.77 | Female | 25 | 2.6 | 19 | 34 | 27 |
| Pancreatic Cancer | 47.51 | Female | 62 | 0 | 19 | 64 | 52 |
| Lung Cancer | 71.13 | Male | 59 | 31.1 | 34 | 57 | 13 |
| Colorectal Cancer | 44.66 | Male | 9 | 5.2 | 19 | 22 | 41 |
| Breast Cancer | 31.67 | Female | 27 | 5.2 | 47 | 64 | 64 |
| Neuroendocrine Tumors | 78.22 | Female | 11 | 1.7 | 5 | 46 | 9 |
| Pancreatic Cancer | 69.33 | Male | 84 |  | 70 | 61 | 89 |
| Uterine Cancer | 68.88 | Female | 45 | 6 | 67 | 72 | 37 |
| Pancreatic Cancer | 72.39 | Male | 38 | 3.4 | 56 | 34 | 47 |
| Colorectal Cancer | 43.45 | Female | 75 |  | 36 | 47 | 77 |
| Pancreatic Cancer | 53.92 | Male | 71 |  | 55 | 68 | 67 |
| Pancreatic Cancer | 74.33 | Female | 37 | 3.4 | 44 | 34 | 27 |
| Small Intestine Cancer | 54.92 | Male | 1 | 2.6 | 31 | 34 | 40 |
| Esophageal Cancer | 78.28 | Male | 23 | 2.6 | 52 | 15 | 68 |
| Pancreatic Cancer | 59.87 | Male | 23 | 2.6 | 35 | 37 | 46 |
| Cervical Cancer | 52.05 | Female | 49 | 3.4 | 61 | 48 | 86 |
| Neuroendocrine Tumors | 64.53 | Female | 35 | 7.8 | 39 | 55 | 49 |
| Colorectal Cancer | 48.57 | Female | 99 | 1.7 | 76 | 73 | 88 |
| Uterine Cancer | 79.77 | Female | 19 | 5.2 | 19 | 12 | 10 |
| Colorectal Cancer | 60.57 | Female | 56 | 3.4 | 31 | 51 | 29 |
| Stomach Cancer | 65.61 | Male | 63 | 5.2 | 82 | 38 | 90 |
| Cervical Cancer | 69.08 | Female | 37 | 4.3 | 56 | 57 | 93 |
| Colorectal Cancer | 67.18 | Female | 64 | 5.2 | 82 | 85 | 89 |
| Stomach Cancer | 35 | Male | 41 |  | 59 | 72 | 83 |
| Pancreatic Cancer | 63.69 | Male | 90 |  | 62 | 69 | 72 |
| Stomach Cancer | 52.54 | Female | 79 | 1.7 | 47 | 93 | 95 |
| Ovarian Cancer | 56.76 | Female | 27 | 4.3 | 7 | 9 | 9 |
| Esophageal Cancer | 84.84 | Male | 10 | 6.1 | 23 | 27 | 42 |
| Pancreatic Cancer | 61.29 | Female | 54 | 2.6 | 14 | 35 | 23 |
| Pancreatic Cancer | 65.68 | Male | 64 | 4.3 | 54 | 82 | 63 |
| Sarcoma | 23.87 | Male | 37 | 6 | 37 | 15 | 40 |
| Esophageal Cancer | 71.12 | Male | 37 | 22.8 | 14 | 72 | 48 |
| Sarcoma | 32.93 | Female | 32 | 0.8 | 19 | 5 | 0 |
| Ovarian Cancer | 34.02 | Female | 63 | 3.5 | 47 | 23 | 47 |
| Liver and Bile Duct Cancer | 64.94 | Male | 48 | 3.4 | 78 | 45 | 71 |
| Uterine Cancer | 61.1 | Female | 11 | 3.4 | 18 | 9 | 33 |
| Colorectal Cancer | 44.68 | Male | 30 | 4.3 | 38 | 9 | 38 |
| Colorectal Cancer | 47.04 | Female | 92 | 4.3 | 90 | 79 | 82 |
| Uterine Cancer | 66.98 | Female | 18 |  | 17 | 8 | 26 |
| Stomach Cancer | 77.68 | Male | 57 | 9.6 | 18 | 11 | 57 |
| Breast Cancer | 49.11 | Female | 78 | 1.7 | 79 | 80 | 84 |
| Colorectal Cancer | 72.13 | Female | 9 |  | 14 | 43 | 9 |
| Colorectal Cancer | 53 | Female | 20 | 8.7 | 29 | 8 | 36 |
| Breast Cancer | 49.33 | Female | 30 | 6.1 | 55 | 26 | 64 |
| Pancreatic Cancer | 31.06 | Female | 72 | 5.2 | 43 | 42 | 41 |
| Uterine Cancer | 55.17 | Female | 100 |  | 67 | 43 | 67 |
| Breast Cancer | 46.66 | Female | 2 | 4.3 | 2 | 3 | 3 |
| Colorectal Cancer | 60.69 | Male | 21 | 2.6 | 10 | 42 | 21 |
| Colorectal Cancer | 69.45 | Male | 27 | 7.8 | 21 | 7 | 44 |
| Lung Cancer | 74.19 | Female | 65 | 6.1 | 80 | 94 | 73 |
| Small Intestine Cancer | 54.4 | Female | 72 | 5.2 | 46 | 44 | 43 |
| Colorectal Cancer | 62.96 | Female | 16 | 2.6 | 50 | 24 | 45 |
| Bladder Cancer | 71.57 | Male | 50 | 4.4 | 68 | 39 | 76 |
| Stomach Cancer | 49.66 | Male | 32 | 4.3 | 59 | 63 | 29 |
| Ovarian Cancer | 77.14 | Female | 49 | 4.4 | 69 | 42 | 68 |
| Pancreatic Cancer | 48.41 | Male | 75 | 2.6 | 73 | 72 | 76 |
| Uterine Cancer | 59.48 | Female | 21 | 3.4 | 48 | 43 | 67 |
| Colorectal Cancer | 56.36 | Female | 81 |  | 90 | 48 | 96 |
| Colorectal Cancer | 43.77 | Male | 18 | 4.3 | 5 | 20 | 8 |
| Colorectal Cancer | 82.3 | Female | 9 |  | 24 | 19 | 53 |
| Liver and Bile Duct Cancer | 93.33 | Female | 0 | 3.4 | 26 | 23 | 0 |
| Squamous Cell Carcinoma of the Skin | 61.94 | Female | 46 |  | 69 | 86 | 64 |
| Head and Neck Cancer | 75.44 | Male | 29 | 1.7 | 23 | 10 | 30 |
| Colorectal Cancer | 49.54 | Male | 31 | 6.9 | 28 | 18 | 39 |
| Colorectal Cancer | 43.96 | Female | 17 | 2.6 | 34 | 28 | 74 |
| Ovarian Cancer | 70.7 | Female | 51 | 5.2 | 95 | 0 | 88 |
| Colorectal Cancer | 42.81 | Female | 30 | 7.8 | 48 | 28 | 82 |
| Cervical Cancer | 56.87 | Female | 86 | 8.7 | 91 | 95 | 83 |
| Breast Cancer | 55.45 | Female | 87 |  | 98 | 89 | 99 |
| Liver and Bile Duct Cancer | 70.85 | Male | 86 | 2.6 | 34 | 68 | 67 |
| Head and Neck Cancer | 67.82 | Female | 27 | 4.3 | 1 | 1 | 9 |
| Neuroendocrine Tumors | 74.77 | Male | 9 | 6.1 | 27 | 57 | 37 |
| Pancreatic Cancer | 57.04 | Male | 53 | 0.8 | 18 | 58 | 50 |
| Mesothelioma | 39.85 | Male | 74 | 0.8 | 79 | 15 | 50 |
| Ovarian Cancer | 71.99 | Female | 83 | 6.1 | 67 | 83 | 68 |
| Esophageal Cancer | 55.33 | Male | 9 |  | 33 | 20 | 31 |
| Colorectal Cancer | 65.52 | Female | 25 | 8.7 | 16 | 38 | 36 |
| Colorectal Cancer | 75.98 | Male | 45 | 7 | 14 | 23 | 13 |
| Colorectal Cancer | 50.61 | Male | 7 | 11.3 | 10 | 42 | 29 |
| Breast Cancer | 35.42 | Female | 55 | 2.6 | 44 | 69 | 41 |
| Stomach Cancer | 44.46 | Female | 45 | 6.1 | 59 | 11 | 31 |
| Uterine Cancer | 32.68 | Female | 18 | 2.6 | 48 | 13 | 0 |
| Uterine Cancer | 57.53 | Female | 10 | 4.3 | 70 | 25 | 98 |
| Uterine Cancer | 71.87 | Female | 50 | 4.3 | 86 | 47 | 71 |
| Pancreatic Cancer | 49.12 | Female | 28 | 5.2 | 99 | 17 | 10 |
| Pancreatic Cancer | 62.93 | Male | 92 | 5.4 | 83 | 82 | 72 |
| Colorectal Cancer | 46.97 | Female | 87 | 15.6 | 61 | 51 | 81 |
| Esophageal Cancer | 60.63 | Female | 8 | 2.6 | 28 | 86 | 77 |
| Ovarian Cancer | 48.02 | Female | 62 | 6 | 53 | 12 | 59 |
| Breast Cancer | 45.84 | Female | 72 | 0.8 | 94 | 52 | 96 |
| Pancreatic Cancer | 77.46 | Male | 62 |  | 94 | 68 | 93 |
| Neuroendocrine Tumors | 41.54 | Male | 9 | 3.5 | 24 | 77 | 25 |
| Pancreatic Cancer | 76.4 | Male | 58 | 3.5 | 42 | 72 | 80 |
| Sarcoma | 25.36 | Female | 42 | 1.8 | 0 | 0 | 88 |
| Esophageal Cancer | 69.21 | Male | 32 | 9.5 | 80 | 17 | 24 |
| Colorectal Cancer | 34.52 | Female | 64 | 7.8 | 94 | 92 | 74 |
| Colorectal Cancer | 40.8 | Female | 7 | 14 | 55 | 0 | 0 |
| Melanoma | 45.16 | Male | 37 | 6.1 | 28 | 42 | 36 |
| Pancreatic Cancer | 75.32 | Female | 16 | 5.2 | 3 | 26 | 16 |
| Colorectal Cancer | 28.06 | Male | 16 | 2.6 | 0 | 0 | 0 |
| Lung Cancer | 71.3 | Female | 30 | 7.9 | 19 | 62 | 18 |
| Colorectal Cancer | 44.39 | Female | 93 |  | 82 | 92 | 71 |
| Unknown Primary Cancer | 70.65 | Female | 62 | 3.4 | 39 | 34 | 21 |
| Colorectal Cancer | 52.13 | Female | 0 |  | 7 | 8 | 2 |
| Small Intestine Cancer | 73.47 | Female | 82 | 2.6 | 84 | 66 | 90 |
| Uterine Cancer | 69.11 | Female | 8 | 5.3 | 7 | 31 | 9 |
| Breast Cancer | 57.26 | Female | 45 | 0.8 | 0 | 4 | 16 |
| Liver and Bile Duct Cancer | 62.18 | Female | 18 |  | 49 | 40 | 83 |
| Colorectal Cancer | 66.88 | Male | 18 | 5.2 | 40 | 10 | 38 |
| Melanoma | 58.21 | Female | 8 | 5.4 | 38 | 5 | 53 |
| Lung Cancer | 61.82 | Male | 45 | 1.7 | 13 | 47 | 53 |
| Colorectal Cancer | 53.59 | Female | 92 | 2.6 | 22 | 35 | 30 |
| Pancreatic Cancer | 55.83 | Male | 55 | 2.7 | 0 | 48 | 0 |
| Colorectal Cancer | 50.7 | Male | 36 | 6.1 | 4 | 85 | 0 |
| Ovarian Cancer | 51.21 | Female | 21 | 7 | 21 | 26 | 29 |
| Ovarian Cancer | 58.15 | Female | 81 | 4.3 | 40 | 47 | 51 |
| Colorectal Cancer | 72.83 | Female | 64 | 3.4 | 98 | 0 | 82 |
| Unknown Primary Cancer | 59.69 | Male | 73 | 3.4 | 24 | 3 | 7 |
| Colorectal Cancer | 65.64 | Male | 18 | 5.3 | 0 | 10 | 65 |
| Colorectal Cancer | 69.06 | Female | 21 |  | 9 | 26 | 18 |
| Lung Cancer | 76.12 | Female | 29 | 8.7 | 32 | 59 | 40 |
| Small Intestine Cancer | 56.62 | Female | 63 |  | 68 | 69 | 76 |
| Colorectal Cancer | 64.05 | Male | 37 | 8.7 | 35 | 27 | 64 |
| Colorectal Cancer | 38.9 | Female | 14 | 4.3 | 40 | 22 | 37 |
| Colorectal Cancer | 64.39 | Female | 40 | 7 | 29 | 37 | 46 |
| Colorectal Cancer | 71.74 | Female | 21 | 31.3 | 50 | 87 | 16 |
| Head and Neck Cancer | 78.18 | Female | 4 | 6 | 15 | 86 | 37 |
| Kidney and Renal Pelvis Cancer | 76.72 | Male | 99 | 5.2 | 100 | 83 | 86 |
| Colorectal Cancer | 73.25 | Male | 9 | 7.8 | 2 | 6 | 6 |
| Colorectal Cancer | 68.28 | Female | 50 | 2.6 | 85 | 87 | 92 |
| Neuroendocrine Tumors | 48.29 | Female | 24 | 11.4 | 22 | 44 | 50 |
| Neuroendocrine Tumors | 61.13 | Female | 11 | 2.6 | 37 | 54 | 33 |
| Liver and Bile Duct Cancer | 59.53 | Female | 50 | 3.5 | 19 | 22 | 2 |
| Prostate Cancer | 53.34 | Male | 7 | 3.5 | 38 | 27 | 36 |
| Colorectal Cancer | 39.29 | Female | 2 | 6.1 | 18 | 35 | 10 |
| Ovarian Cancer | 65.12 | Female | 75 | 1.7 | 89 | 38 | 71 |
| Pancreatic Cancer | 77.51 | Male | 89 | 5.3 | 0 | 61 | 91 |
| Pancreatic Cancer | 74.87 | Female | 48 | 9.6 | 4 | 36 | 19 |
| Colorectal Cancer | 61.09 | Male | 75 | 29.7 | 53 | 61 | 73 |
| Pancreatic Cancer | 38.42 | Female | 46 |  | 73 | 52 | 62 |
| Colorectal Cancer | 38.52 | Female | 12 | 4.3 | 18 | 13 | 24 |
| Colorectal Cancer | 61.87 | Male | 39 | 7.8 | 7 | 11 | 8 |
| Unknown Primary Cancer | 63.55 | Male | 6 | 1.7 | 4 | 8 | 9 |
| Colorectal Cancer | 54.31 | Male | 18 | 6.9 | 41 | 14 | 54 |
| Uterine Cancer | 59.18 | Female | 13 | 2.6 | 86 | 87 | 30 |
| Endocrine Gland Cancer | 53.46 | Female | 34 | 1.7 | 13 | 34 | 9 |
| Pancreatic Cancer | 71.82 | Male | 32 | 2.6 | 0 | 0 | 29 |
| Colorectal Cancer | 75.06 | Male | 56 | 2.6 | 65 | 51 | 72 |
| Colorectal Cancer | 54.68 | Male | 15 | 6.1 | 48 | 27 | 70 |
| Colorectal Cancer | 56.39 | Female | 16 | 4.4 | 6 | 5 | 0 |
| Breast Cancer | 56.42 | Female | 38 | 7 | 18 | 5 | 16 |
| Breast Cancer | 45.33 | Female | 36 | 5.2 | 52 | 26 | 42 |
| Ovarian Cancer | 83.18 | Female | 4 | 3.4 | 38 | 4 | 25 |
| Sarcoma | 68.84 | Female | 6 | 2.6 | 15 | 16 | 6 |
| Brain and Nervous System Cancer | 65.72 | Female | 80 | 1.7 | 1 | 21 | 20 |
| Pancreatic Cancer | 67.94 | Male | 78 | 3.4 | 83 | 75 | 83 |
| Colorectal Cancer | 58.54 | Female | 59 | 4.3 | 0 | 38 | 64 |
| Mesothelioma | 79.56 | Male | 94 | 1.7 | 62 | 50 | 44 |
| Pancreatic Cancer | 69.08 | Male | 62 |  | 84 | 63 | 78 |
| Colorectal Cancer | 78.31 | Male | 51 | 21.7 | 15 | 41 | 25 |
| Colorectal Cancer | 49.73 | Female | 49 | 4.3 | 7 | 22 | 30 |
| Colorectal Cancer | 44.1 | Male | 74 |  | 82 | 35 | 44 |
| Breast Cancer | 53.8 | Female | 44 | 5.2 | 24 | 22 | 11 |
| Colorectal Cancer | 67.34 | Female | 33 | 8.7 | 73 | 44 | 53 |
| Ovarian Cancer | 57.7 | Female | 38 | 4.3 | 14 | 87 | 22 |
| Pancreatic Cancer | 73.26 | Female | 90 | 2.6 | 82 | 62 | 81 |
| Ovarian Cancer | 70.14 | Female | 70 | 2.6 | 62 | 54 | 64 |
| Lung Cancer | 63.84 | Male | 60 | 11.3 | 54 | 99 | 54 |
| Colorectal Cancer | 53.99 | Male | 65 | 5.2 | 13 | 51 | 30 |
| Unknown Primary Cancer | 77.42 | Female | 80 | 3.5 | 69 | 34 | 31 |
| Uterine Cancer | 57.21 | Female | 16 | 6.1 | 59 | 28 | 64 |
| Kidney and Renal Pelvis Cancer | 74.25 | Male | 91 | 5.2 | 74 | 65 | 30 |
| Breast Cancer | 66.39 | Female | 85 | 4.7 | 41 | 55 | 12 |
| Esophageal Cancer | 67.17 | Male | 7 | 0.8 | 11 | 35 | 27 |
| Pancreatic Cancer | 69.84 | Female | 92 | 2.6 | 78 | 69 | 83 |
| Unknown Primary Cancer | 61.79 | Male | 2 | 0.8 | 5 | 2 | 2 |
| Colorectal Cancer | 68.8 | Unspecified | 70 | 9.6 | 25 | 28 | 11 |
| Breast Cancer | 63.99 | Female | 80 | 0.8 | 68 | 34 | 49 |
| Pancreatic Cancer | 74.59 | Male | 96 | 5.2 | 82 | 68 | 90 |
| Uterine Cancer | 64.46 | Female | 78 | 6.1 | 50 | 54 | 30 |
| Colorectal Cancer | 51.92 | Male | 22 | 2.6 | 5 | 33 | 18 |
| Esophageal Cancer | 45.52 | Female | 58 | 10.3 | 64 | 68 | 47 |
| Prostate Cancer | 57.88 | Male | 24 | 0 | 21 | 10 | 12 |
| Sarcoma | 65.17 | Male | 18 | 2.6 | 36 | 9 | 20 |
| Colorectal Cancer | 50.49 | Female | 40 | 8.7 | 58 | 0 | 42 |
| Ovarian Cancer | 40.58 | Female | 16 | 5.2 | 15 | 2 | 8 |
| Ovarian Cancer | 50.83 | Female | 32 | 4.3 | 45 | 71 | 32 |
| Pancreatic Cancer | 73.9 | Male | 61 |  | 92 | 77 | 87 |
| Pancreatic Cancer | 63.53 | Female | 0 |  | 0 | 51 | 0 |
| Colorectal Cancer | 61.97 | Male | 19 | 10.4 | 13 | 9 | 24 |
| Sarcoma | 56.64 | Male | 56 | 1.7 | 38 | 79 | 32 |
| Colorectal Cancer | 57.22 | Female | 87 |  | 26 | 34 | 4 |
| Sarcoma | 71.03 | Male | 78 |  | 91 | 68 | 89 |
| Ovarian Cancer | 77.66 | Female | 35 | 1.7 | 27 | 26 | 13 |
| Pancreatic Cancer | 84.55 | Female | 94 | 6 | 68 | 87 | 78 |
| Colorectal Cancer | 47.54 | Female | 54 | 5.2 | 35 | 8 | 4 |
| Ovarian Cancer | 31.34 | Female | 16 | 3.4 | 51 | 6 | 30 |
| Breast Cancer | 55.37 | Female | 58 | 8.7 | 92 | 86 | 85 |
| Liver and Bile Duct Cancer | 72.2 | Male | 51 |  | 75 | 47 | 9 |
| Neuroendocrine Tumors | 70.66 | Female | 65 | 9.6 | 81 | 78 | 100 |
| Breast Cancer | 60.47 | Female | 77 | 1.7 | 32 | 24 | 40 |
| Esophageal Cancer | 52.6 | Male | 21 | 1.7 | 26 | 26 | 50 |
| Neuroendocrine Tumors | 58.27 | Male | 96 | 3.5 | 100 | 93 | 95 |
| Ovarian Cancer | 58.67 | Female | 28 | 6.1 | 33 | 22 | 16 |
| Sarcoma | 53.98 | Male | 37 | 2.6 | 5 | 24 | 33 |
| Breast Cancer | 42.56 | Female | 77 | 0.8 | 48 | 75 | 38 |
| Colorectal Cancer | 36.24 | Female | 23 | 8.7 | 35 | 26 | 57 |
| Colorectal Cancer | 65.62 | Male | 72 | 5.2 | 14 | 40 | 24 |
| Colorectal Cancer | 49.38 | Female | 88 | 2.6 | 24 | 12 | 10 |
| Colorectal Cancer | 65.97 | Female | 30 | 6.1 | 73 | 86 | 57 |
| Esophageal Cancer | 70.37 | Male | 28 | 4.3 | 58 | 77 | 67 |
| Breast Cancer | 47.76 | Female | 44 | 5.2 | 76 | 34 | 44 |
| Colorectal Cancer | 56.87 | Male | 84 | 0.8 | 67 | 42 | 77 |
| Neuroendocrine Tumors | 64.55 | Male | 4 | 3.5 | 0 | 34 | 10 |
| Colorectal Cancer | 83.21 | Female | 41 | 5.2 | 24 | 13 | 23 |
| Ovarian Cancer | 71.86 | Female | 64 | 5.2 | 91 | 42 | 36 |
| Breast Cancer | 39.23 | Female | 21 | 0.8 | 8 | 6 | 97 |
| Colorectal Cancer | 46.7 | Male | 12 |  | 19 | 16 | 65 |
| Colorectal Cancer | 64.16 | Male | 21 | 6.1 | 36 | 6 | 29 |
| Breast Cancer | 31.26 | Female | 17 | 2.6 | 22 | 6 | 4 |
| Colorectal Cancer | 60.04 | Female | 45 | 2.6 | 75 | 60 | 81 |
| Colorectal Cancer | 76.96 | Male | 4 | 4.3 | 23 | 12 | 29 |
| Breast Cancer | 62.08 | Female | 41 | 0.8 | 63 | 53 | 44 |
| Bladder Cancer | 62.17 | Male | 50 | 5.2 | 36 | 38 | 37 |
| Colorectal Cancer | 59.39 | Male | 9 | 4.3 | 2 | 3 | 8 |
| Colorectal Cancer | 57.67 | Male | 14 | 12.2 | 26 | 8 | 26 |
| Gallbladder and Extrahepatic Bile Duct Cancers | 51.48 | Female | 21 | 3.5 | 18 | 34 | 21 |
| Pancreatic Cancer | 45.55 | Female | 37 | 7 | 6 | 9 | 6 |
| Colorectal Cancer | 66.12 | Male | 25 | 3.4 | 39 | 26 | 11 |
| Melanoma | 82.75 | Male | 85 | 17.5 | 97 | 78 | 81 |
| Stomach Cancer | 32.11 | Male | 19 | 3.4 | 28 | 44 | 65 |
| Stomach Cancer | 66.64 | Female | 35 | 4.3 | 68 | 91 | 42 |
| Colorectal Cancer | 52.27 | Female | 37 | 4.3 | 18 | 10 | 28 |
| Uterine Cancer | 70.01 | Female | 5 | 0.8 | 59 | 32 | 45 |
| Sarcoma | 86.15 | Male | 57 | 1.7 | 23 | 26 | 24 |
| Ovarian Cancer | 32.13 | Female | 96 | 9.7 | 65 | 38 | 57 |
| Breast Cancer | 43.46 | Female | 84 | 8.9 | 37 | 34 | 54 |
| Colorectal Cancer | 45.79 | Male | 83 | 33.7 | 79 | 73 | 61 |
| Liver and Bile Duct Cancer | 53.94 | Male | 4 | 4.4 | 5 | 4 | 5 |
| Pancreatic Cancer | 55.55 | Female | 50 | 0 | 34 | 26 | 5 |
| Colorectal Cancer | 41.17 | Male | 26 | 1.7 | 24 | 10 | 29 |
| Colorectal Cancer | 59.11 | Female | 28 | 2.6 | 59 | 54 | 67 |
| Colorectal Cancer | 30.76 | Male | 21 | 4.3 | 45 | 8 | 50 |
| Unknown Primary Cancer | 89.22 | Female | 18 | 11.3 | 51 | 62 | 42 |
| Colorectal Cancer | 51.98 | Male | 83 |  | 83 | 87 | 95 |
| Colorectal Cancer | 37.25 | Female | 62 | 20.1 | 94 | 74 | 72 |
| Kidney and Renal Pelvis Cancer | 53.13 | Female | 48 | 5.2 | 50 | 8 | 10 |
| Colorectal Cancer | 36.18 | Female | 18 | 4.4 | 18 | 6 | 0 |
| Pancreatic Cancer | 80.54 | Female | 69 | 3.4 | 53 | 39 | 46 |
| Lipomatous Neoplasms | 78.65 | Female | 65 | 2.6 | 4 | 44 | 20 |
| Lung Cancer | 68.69 | Male | 0 | 4.4 | 11 | 1 | 0 |
| Sarcoma | 34.37 | Female | 40 | 1.7 | 8 | 16 | 64 |
| Liver and Bile Duct Cancer | 65.77 | Male | 62 | 3.5 | 73 | 38 | 57 |
| Breast Cancer | 67.35 | Female | 63 | 2.6 | 22 | 8 | 8 |
| Uterine Cancer | 70.08 | Female | 0 | 4.3 | 43 | 15 | 0 |
| Colorectal Cancer | 70.8 | Male | 60 | 1.7 | 89 | 35 | 93 |
| Breast Cancer | 51.79 | Female | 87 | 2.6 | 92 | 95 | 96 |
| Colorectal Cancer | 52.96 | Female | 21 | 7 | 36 | 7 | 65 |
| Neuroendocrine Tumors | 58.34 | Male | 32 | 1.7 | 9 | 27 | 22 |
| Neuroendocrine Tumors | 40.71 | Male | 28 | 1.7 | 21 | 20 | 2 |
| Uterine Cancer | 88.56 | Female | 42 |  | 79 | 61 | 64 |
| Colorectal Cancer | 64.19 | Male | 26 | 7.9 | 15 | 20 | 42 |
| Colorectal Cancer | 57.79 | Female | 6 | 4.4 | 41 | 24 | 43 |
| Colorectal Cancer | 59.63 | Female | 29 | 5.2 | 79 | 68 | 78 |
| Esophageal Cancer | 62.53 | Female | 35 | 0.8 | 83 | 60 | 67 |
| Breast Cancer | 32.17 | Female | 96 | 2.6 | 47 | 67 | 60 |
| Stomach Cancer | 59.74 | Male | 54 | 6.1 | 21 | 26 | 20 |
| Colorectal Cancer | 64.53 | Male | 35 | 3.5 | 41 | 41 | 43 |
| Esophageal Cancer | 86.39 | Male | 29 | 17.4 | 58 | 73 | 43 |
| Stomach Cancer | 32.77 | Male | 43 | 1.7 | 22 | 48 | 47 |
| Breast Cancer | 49.91 | Female | 53 | 4.3 | 55 | 30 | 45 |
| Uterine Cancer | 76.13 | Female | 15 | 3.5 | 24 | 24 | 60 |
| Colorectal Cancer | 54.39 | Male | 35 | 7 | 12 | 24 | 4 |
| Breast Cancer | 38.46 | Female | 48 | 6 | 27 | 7 | 3 |
| Pancreatic Cancer | 56.23 | Female | 56 | 3.4 | 74 | 64 | 30 |
| Pancreatic Cancer | 68.02 | Female | 11 | 3.5 | 5 | 3 | 6 |
| Colorectal Cancer | 66.84 | Male | 36 |  | 35 | 87 | 47 |
| Liver and Bile Duct Cancer | 80.72 | Female | 17 | 6.1 | 33 | 0 | 68 |
| Head and Neck Cancer | 40.07 | Male | 12 | 0 | 10 | 40 | 7 |
| Ovarian Cancer | 75.26 | Female | 12 | 3.4 | 9 | 6 | 9 |
| Colorectal Cancer | 62 | Male | 18 | 6.1 | 20 | 44 | 53 |
| Colorectal Cancer | 59.25 | Male | 37 |  | 89 | 45 | 69 |
| Colorectal Cancer | 78.05 | Female | 37 | 79.9 | 58 | 60 | 30 |
| Sarcoma | 73.32 | Female | 4 | 4.3 | 22 | 56 | 21 |
| Unknown Primary Cancer | 74.82 | Female | 52 |  | 16 | 53 | 0 |
| Pancreatic Cancer | 67.33 | Male | 77 | 4.3 | 48 | 76 | 48 |
| Esophageal Cancer | 66.99 | Male | 17 | 1.7 | 53 | 26 | 54 |
| Breast Cancer | 76.14 | Female | 48 | 4.4 | 68 | 40 | 79 |
| Breast Cancer | 38.96 | Female | 45 | 7.9 | 67 | 60 | 0 |
| Stomach Cancer | 66.12 | Female | 53 |  | 0 | 40 | 47 |
| Neuroendocrine Tumors | 51.39 | Male | 12 | 61 | 19 | 4 | 14 |
| Colorectal Cancer | 77.54 | Male | 29 |  | 37 | 41 | 64 |
| Pancreatic Cancer | 66.05 | Male | 62 | 4.3 | 69 | 84 | 33 |
| Lung Cancer | 57.11 | Female | 83 | 6.9 | 35 | 60 | 30 |
| Ovarian Cancer | 79.99 | Female | 32 | 1.7 | 54 | 11 | 30 |
| Sarcoma | 51.71 | Female | 18 | 5.2 | 46 | 0 | 0 |
| Brain and Nervous System Cancer | 52.61 | Female | 52 | 2.6 | 41 | 22 | 27 |
| Colorectal Cancer | 60.42 | Male | 13 | 6 | 7 | 10 | 10 |
| Gallbladder and Extrahepatic Bile Duct Cancers | 55.1 | Female | 29 | 8.7 | 31 | 11 | 7 |
| Unknown Primary Cancer | 68.84 | Female | 31 | 3.4 | 0 | 18 | 2 |
| Stomach Cancer | 28.74 | Female | 8 | 1.7 | 47 | 21 | 17 |
| Breast Cancer | 54.11 | Female | 64 | 2.6 | 74 | 40 | 81 |
| Colorectal Cancer | 47.08 | Male | 29 | 5.2 | 25 | 13 | 18 |
| Colorectal Cancer | 31.77 | Female | 12 | 1.7 | 19 | 24 | 27 |
| Sarcoma | 72.26 | Male | 81 | 1.7 | 67 | 72 | 71 |
| Head and Neck Cancer | 68.13 | Male | 12 | 3.4 | 18 | 5 | 18 |
| Head and Neck Cancer | 50.4 | Male | 45 | 5.2 | 89 | 56 | 95 |
| Colorectal Cancer | 51.63 | Female | 3 | 4.3 | 35 | 15 | 30 |
| Uterine Cancer | 51.47 | Female | 16 | 3.4 | 53 | 13 | 33 |
| Small Intestine Cancer | 62.78 | Female | 45 | 8.6 | 53 | 67 | 74 |
| Breast Cancer | 46.71 | Female | 9 | 5.2 | 27 | 2 | 20 |
| Ovarian Cancer | 63.26 | Female | 32 | 3.5 | 67 | 15 | 19 |
| Lipomatous Neoplasms | 60.17 | Male | 53 | 1.7 | 28 | 12 | 70 |
| Colorectal Cancer | 65.15 | Female | 32 | 2.6 | 76 | 53 | 71 |
| Lung Cancer | 67.18 | Female | 65 | 2.6 | 46 | 37 | 44 |
| Stomach Cancer | 46.05 | Male | 1 | 3.4 | 77 | 55 | 57 |
| Colorectal Cancer | 66.38 | Female | 55 | 5.2 | 83 | 79 | 69 |
| Neuroendocrine Tumors | 78.61 | Female | 10 | 3.4 | 8 | 3 | 8 |
| Pancreatic Cancer | 72.51 | Male | 71 | 6.9 | 38 | 86 | 5 |
| Breast Cancer | 53.95 | Female | 46 | 7.8 | 9 | 18 | 8 |
| Pancreatic Cancer | 59.76 | Male | 79 | 6.1 | 79 | 50 | 68 |
| Breast Cancer | 72.76 | Female | 0 | 3.5 | 30 | 1 | 9 |
| Lung Cancer | 62.28 | Male | 52 | 6 | 72 | 64 | 69 |
| Small Intestine Cancer | 53.07 | Male | 68 | 5.2 | 94 | 70 | 91 |
| Liver and Bile Duct Cancer | 47.01 | Female | 75 | 5.2 | 42 | 48 | 38 |
| Melanoma | 73.47 | Female | 92 | 9.5 | 97 | 80 | 80 |
| Uterine Cancer | 67.24 | Female | 10 | 5.2 | 14 | 3 | 4 |
| Small Intestine Cancer | 64.75 | Female | 27 | 6.9 | 45 | 62 | 26 |
| Colorectal Cancer | 77.26 | Female | 57 | 1.7 | 19 | 15 | 15 |
| Small Intestine Cancer | 63.64 | Male | 43 | 4.4 | 46 | 35 | 71 |
| Breast Cancer | 60.9 | Female | 15 | 3.5 | 5 | 18 | 0 |
| Colorectal Cancer | 55.76 | Female | 55 | 2.6 | 14 | 9 | 19 |
| Head and Neck Cancer | 29.05 | Male | 46 | 2.6 | 22 | 86 | 24 |
| Breast Cancer | 48.56 | Female | 37 | 1.7 | 54 | 30 | 38 |
| Colorectal Cancer | 27.71 | Male | 69 | 6 | 58 | 62 | 30 |
| Stomach Cancer | 33.22 | Male | 37 | 3.4 | 80 | 74 | 73 |
| Breast Cancer | 47.76 | Female | 83 |  | 95 | 67 | 78 |
| Colorectal Cancer | 66.42 | Male | 40 | 2.6 | 85 | 46 | 47 |
| Ovarian Cancer | 57.05 | Female | 45 | 5.2 | 67 | 81 | 58 |
| Lung Cancer | 71.35 | Female | 64 |  | 0 | 12 | 0 |
| Breast Cancer | 69.87 | Female | 52 | 4.3 | 40 | 26 | 38 |
| Pancreatic Cancer | 56.79 | Male | 12 | 4.3 | 22 | 27 | 15 |
| Pancreatic Cancer | 74.65 | Female | 86 | 0.8 | 73 | 62 | 64 |
| Liver and Bile Duct Cancer | 68.81 | Male | 45 | 5.2 | 15 | 41 | 0 |
| Colorectal Cancer | 84.39 | Female | 8 | 8.7 | 25 | 8 | 0 |
| Colorectal Cancer | 78.02 | Male | 30 | 4.3 | 89 | 17 | 76 |
| Unknown Primary Cancer | 76.39 | Male | 27 | 3.5 | 50 | 0 | 0 |
| Colorectal Cancer | 47.32 | Female | 37 | 3.5 | 55 | 17 | 0 |
| Squamous Cell Carcinoma of the Skin | 76.73 | Female | 25 | 2.6 | 12 | 80 | 51 |
| Unknown Primary Cancer | 60.01 | Female | 93 | 7 | 75 | 85 | 77 |
| Unknown Primary Cancer | 59.2 | Male | 3 | 4.3 | 44 | 14 | 7 |
| Colorectal Cancer | 63.69 | Female | 2 | 1.7 | 52 | 42 | 59 |
| Pancreatic Cancer | 61.69 | Male | 83 | 2.6 | 34 | 20 | 19 |
| Pancreatic Cancer | 53.34 | Male | 42 | 5.2 | 0 | 26 | 32 |
| Ovarian Cancer | 70.86 | Female | 96 | 3.4 | 95 | 94 | 84 |
| Ovarian Cancer | 43.52 | Female | 29 | 2.6 | 56 | 41 | 15 |
| Squamous Cell Carcinoma of the Skin | 46.22 | Female | 52 | 6.1 | 30 | 69 | 57 |
| Thyroid Cancer | 66.52 | Female | 31 | 7 | 2 | 66 | 81 |
| Ovarian Cancer | 64.22 | Female | 63 | 3.4 | 80 | 58 | 69 |
| Stomach Cancer | 60.03 | Female | 32 | 6.3 | 41 | 35 | 42 |
| Brain and Nervous System Cancer | 56.37 | Male | 79 | 2.6 | 25 | 14 | 8 |
| Ovarian Cancer | 63.37 | Female | 44 | 0.8 | 52 | 33 | 9 |
| Liver and Bile Duct Cancer | 61.63 | Female | 74 |  | 91 | 0 | 0 |
| Prostate Cancer | 77.31 | Male | 30 | 2.6 | 14 | 3 | 2 |
| Small Intestine Cancer | 55.47 | Male | 87 |  | 44 | 30 | 35 |
| Breast Cancer | 44.79 | Female | 20 | 0.8 | 23 | 28 | 25 |
| Colorectal Cancer | 53.92 | Male | 12 | 4.3 | 59 | 47 | 85 |
| Colorectal Cancer | 50.28 | Female | 47 | 5.2 | 46 | 38 | 26 |
| Breast Cancer | 56.04 | Female | 55 | 4.4 | 16 | 10 | 9 |
| Colorectal Cancer | 68.08 | Female | 44 | 3.4 | 95 | 9 | 72 |
| Lung Cancer | 76.06 | Male | 31 |  | 0 | 22 | 0 |
| Stomach Cancer | 64.6 | Male | 39 | 5.2 | 53 | 57 | 29 |
| Ovarian Cancer | 69.99 | Female | 8 | 2.6 | 8 | 10 | 7 |
| Lung Cancer | 40.41 | Male | 78 |  | 42 | 17 | 44 |
| Colorectal Cancer | 52.44 | Female | 9 | 6.2 | 22 | 47 | 68 |
| Colorectal Cancer | 48 | Male | 28 | 8.7 | 16 | 48 | 39 |
| Unknown Primary Cancer | 70 | Male | 18 | 32.2 | 8 | 68 | 23 |
| Stomach Cancer | 74.41 | Male | 5 | 6.1 | 19 | 2 | 19 |
| Sarcoma | 38.56 | Female | 21 | 10.5 | 33 | 55 | 10 |
| Ovarian Cancer | 78.97 | Female | 63 | 6.1 | 42 | 49 | 57 |
| Sarcoma | 29.47 | Male | 37 | 2.6 | 34 | 32 | 28 |
| Ovarian Cancer | 66.78 | Female | 0 | 2.6 | 3 | 0 | 2 |
| Stomach Cancer | 56.99 | Male | 40 | 6.1 | 40 | 8 | 38 |
| Breast Cancer | 35.95 | Female | 71 | 5.2 | 29 | 40 | 81 |
| Ovarian Cancer | 64.87 | Female | 25 |  | 48 | 23 | 12 |
| Thyroid Cancer | 53.08 | Female | 79 | 4.3 | 95 | 86 | 97 |
| Stomach Cancer | 48.7 | Female | 47 | 3.5 | 40 | 85 | 64 |
| Breast Cancer | 62.39 | Female | 26 | 3.5 | 8 | 4 | 16 |
| Melanoma | 67.17 | Male | 22 | 1.7 | 49 | 20 | 27 |
| Breast Cancer | 72.46 | Female | 82 | 6.1 | 92 | 86 | 91 |
| Pancreatic Cancer | 43.34 | Female | 81 |  | 92 | 12 | 69 |
| Colorectal Cancer | 77.94 | Female | 41 | 3.4 | 82 | 61 | 71 |
| Liver and Bile Duct Cancer | 66.48 | Male | 58 |  | 77 | 5 | 51 |

Notes: *percentile rank of RNA expression. Blank boxes are places where we do not have this patient data.
